# Multidimensional Longitudinal Assessment of Oral Mucositis Burden and Functional Impact in Head and Neck Cancer Patients Undergoing Radiotherapy or Chemoradiotherapy: A Retrospective and Exploratory Observational Study

**DOI:** 10.3390/cancers18132076

**Published:** 2026-06-26

**Authors:** Bianca Santo, Matteo Romanello, Paola De Franco, Elisa Cavalera, Donatella Russo, Dino Rubini, Antonio Palumbo, Giuseppe Rubini, Angela Sardaro

**Affiliations:** 1 Radiotherapy, “Vito Fazzi” Hospital, 73100 Lecce, Italy; 2 Otorhinolaryngology, Head and Neck Surgery, “Vito Fazzi” Hospital, 73100 Lecce, Italy; 3 Radiation Therapy Unit, Department of Precision Medicine, Università degli Studi della Campania Luigi Vanvitelli, 80129 Napoli, Italy; 4 Nuclear Medicine Unit, Interdisciplinary Department of Medicine, University of Bari, 70124 Bari, Italy

**Keywords:** head and neck cancer, supportive therapies, oral mucositis, MDADI, OMAS, CTCAE

## Abstract

**Background/Objectives**: Oral mucositis is a frequent and clinically significant acute toxicity in patients undergoing radiotherapy or chemoradiotherapy for head and neck cancer, with substantial consequences for swallowing function, nutritional status, and quality of life. Conventional clinician-reported toxicity grading may not fully capture the multidimensional burden experienced by patients. This study aimed to perform a longitudinal multidimensional assessment of treatment-related oral mucositis by evaluating the relationship between clinician-reported toxicity, objective mucosal injury, patient-reported swallowing-related quality of life, and nutritional status. **Methods**: In this retrospective observational study, 32 of 54 consecutively screened patients with locally advanced head and neck cancer treated with curative-intent radiotherapy, with or without concurrent chemotherapy, were included. Oral mucositis was assessed using the Common Terminology Criteria for Adverse Events (CTCAE) and the Oral Mucositis Assessment Scale (OMAS). Swallowing-related quality of life was evaluated using the MD Anderson Dysphagia Inventory (MDADI). Body weight was recorded longitudinally as an indicator of nutritional status. Correlations between OMAS scores and clinical outcome measures were analyzed at predefined timepoints using Spearman’s rank correlation coefficient. **Results**: All patients developed treatment-related oral mucositis, with peak severity occurring during the acute treatment phase. During the acute treatment phase, OMAS scores demonstrated a moderate positive correlation with clinician-reported toxicity (CTCAE) and inverse correlations with MDADI scores. A significant inverse association between OMAS and MDADI composite score persisted at treatment completion (ρ = −0.41, *p* = 0.022). Body weight progressively declined during treatment, although no statistically significant correlation with OMAS severity was observed. **Conclusions**: A multidimensional assessment integrating clinician-reported toxicity, objective mucosal evaluation, and patient-reported functional outcomes provides a broader characterization of oral mucositis burden in patients undergoing radiotherapy-based treatment for head and neck cancer. These findings support the potential value of integrated toxicity assessment strategies to improve supportive care monitoring in clinical practice.

## Simple Summary

Oral mucositis is a common and painful side effect in patients receiving radiotherapy or chemoradiotherapy for head and neck cancer. It can significantly affect eating, swallowing, and overall quality of life, sometimes leading to treatment interruptions. In clinical practice, mucositis is usually evaluated by physicians, but this may not fully reflect the patient’s experience. In this study, we analyzed different ways of assessing mucositis, including clinical evaluation, direct observation of mucosal damage, and patient-reported outcomes. We also explored how mucositis relates to changes in body weight during treatment. Our findings suggest that combining these different assessments provides a more comprehensive understanding of the impact of mucositis on patients. This approach may help clinicians better identify patients at higher risk of complications and improve supportive care strategies during cancer treatment.

## 1. Introduction

Oral mucositis (OM) is one of the most frequent and clinically relevant acute toxicities in patients undergoing radiotherapy or chemoradiotherapy for head and neck cancer (HNC), with a substantial impact on treatment tolerance, nutritional status, swallowing function, and overall quality of life [1,2,3,4]. The pathobiology of OM is characterized by a complex cascade of biological events involving tissue injury, inflammatory signaling amplification, ulceration, and healing, resulting in clinically significant symptom burden during treatment [4,5,6].

Severe OM may lead to oral pain, impaired oral intake, dysphagia, treatment interruptions, increased supportive care requirements, and, in some cases, reduced treatment adherence [7,8,9,10]. In patients with HNC, where treatment-related toxicity directly affects oral function and swallowing, these complications may have a particularly pronounced impact on both short-term clinical management and patient-reported quality of life [11,12].

In routine oncology practice, the assessment of oral mucositis commonly relies on clinician-reported toxicity grading systems such as the Common Terminology Criteria for Adverse Events (CTCAE). Although these tools remain widely adopted, they may not fully capture the multidimensional burden experienced by patients, particularly with regard to functional impairment and symptom perception [13,14].

Objective mucosal assessment tools, such as the Oral Mucositis Assessment Scale (OMAS), provide a more granular evaluation of visible mucosal injury [15]. In parallel, patient-reported outcome measures are increasingly recognized as essential complementary instruments for evaluating treatment-related toxicity, especially in head and neck oncology, where acute symptoms may substantially affect swallowing, oral intake, communication, and social functioning [16,17].

However, the longitudinal relationship between clinician-reported toxicity, objective mucosal damage, patient-reported functional impairment, and nutritional status remains incompletely characterized in real-world patients undergoing radiotherapy-based treatment for HNC. A broader understanding of these complementary dimensions may contribute to a more comprehensive characterization of treatment-related toxicity and patient experience.

The aim of the present retrospective observational study was to perform a multidimensional longitudinal assessment of treatment-related oral mucositis in patients undergoing radiotherapy or chemoradiotherapy for head and neck cancer by evaluating the relationship between clinician-reported toxicity, objective mucosal findings, patient-reported swallowing-related quality of life, and body weight changes over time.

## 2. Materials and Methods

### 2.1. Study Design and Patient Population

This retrospective observational study included consecutive patients with locally advanced head and neck cancer treated with curative-intent radiotherapy, with or without concurrent chemotherapy, at our institution between March 2025 and January 2026.

**Figure 1:**
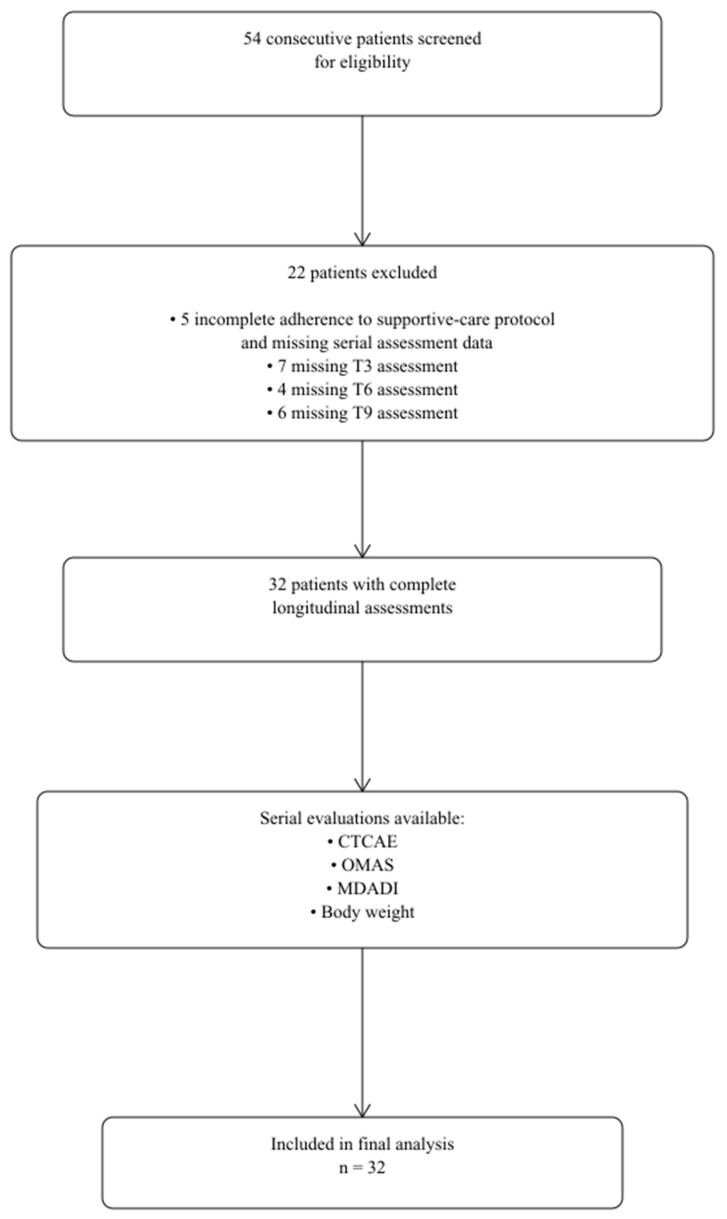
Flow diagram of patient selection and inclusion. A total of 54 consecutive patients treated with radiotherapy-based therapy for head and neck cancer were screened. Twenty-two patients were excluded because complete longitudinal clinical assessments were not available, including five patients with incomplete adherence to the supportive-care protocol resulting in missing serial assessment data, seven patients who missed the scheduled T3 evaluation, four patients who missed the T6 evaluation, and six patients who missed the T9 evaluation. The final study cohort consisted of 32 patients with complete CTCAE, OMAS, MDADI, and body weight data available at all predefined assessment timepoints.

Eligible patients were adults with histologically confirmed head and neck malignancies who completed radiotherapy-based treatment and underwent longitudinal clinical assessment during treatment and follow-up. Patients were included if complete data regarding oral mucositis assessment (CTCAE and OMAS), swallowing-related quality of life (MDADI), and body weight were available at all predefined assessment timepoints. Patients who did not undergo the scheduled serial otorhinolaryngological evaluations during treatment and follow-up, or who lacked complete supportive care monitoring data, were excluded because longitudinal assessment of the study variables could not be reliably performed. Consequently, complete serial OMAS, CTCAE, and MDADI evaluations were not available for these patients. Fifty-four consecutive patients treated with radiotherapy-based treatment were screened for eligibility. Twenty-two patients were excluded because complete longitudinal assessments were not available: five patients started the supportive-care protocol from the second treatment week, seven patients missed the scheduled otorhinolaryngological T3 assessment, four patients missed the scheduled otorhinolaryngological assessment at T6, and six patients missed the scheduled otorhinolaryngological assessment at T9. Therefore, 32 patients with complete serial CTCAE, OMAS, MDADI, and body weight data were included in the final analysis (Figure 1).

Because of the retrospective exploratory nature of the study, no formal sample size calculation was performed.

### 2.2. Treatment and Supportive Care

All patients underwent radiotherapy delivered using volumetric modulated arc therapy (VMAT) according to institutional treatment protocols. Treatment was delivered either in the definitive or adjuvant setting, with concurrent systemic therapy administered when clinically indicated according to multidisciplinary treatment planning. Prescribed doses to the high-risk target volume ranged from 60 to 70 Gy, depending on disease site, stage, and treatment setting. Concurrent systemic treatment consisted predominantly of weekly cisplatin, while one patient received weekly cetuximab.

Throughout treatment, all patients received supportive care according to routine institutional clinical practice for the prevention and management of treatment-related oral toxicities. This included oral hygiene recommendations, symptomatic supportive management, nutritional monitoring, and pharmacological interventions when clinically indicated. Kariosyte^®^ was routinely prescribed as part of institutional supportive oral care from the second week of treatment. The product contains compounds with hydrating, barrier-forming, antioxidant, and soothing properties that may support oral mucosal care during radiotherapy. However, the present study was not designed to evaluate its efficacy.

### 2.3. Clinical Assessment

Patients underwent regular clinical evaluation as part of standard care.

Clinician-reported toxicity was assessed by the treating radiation oncologist using the Common Terminology Criteria for Adverse Events (CTCAE, version 5.0) during routine weekly evaluations. Objective mucosal assessment (OMAS) and patient-reported swallowing-related quality-of-life evaluation using the MD Anderson Dysphagia Inventory (MDADI) were performed during scheduled otorhinolaryngological follow-up visits by experienced head and neck specialists. All assessments were conducted according to standardized institutional clinical practice. Because of the retrospective nature of the study, formal inter-rater reliability testing was not available. OMAS scoring was based on the evaluation of predefined oral and oropharyngeal mucosal sites, assessing the severity of mucosal injury according to the original OMAS methodology. Endoscopic findings were used for routine clinical documentation and illustrative purposes but were not used as the primary basis for OMAS scoring. The Italian validated version of the MDADI questionnaire was administered at each predefined assessment timepoint. The global score was derived from the single global item (range 1–5), whereas the composite score was calculated according to the original MDADI scoring system and expressed on a 0–100 scale, with higher scores indicating better swallowing-related quality of life.

Body weight was recorded longitudinally as an indicator of nutritional status and treatment-related functional burden.

Assessments were performed at predefined weekly timepoints throughout treatment and follow-up:

- T0: baseline assessment before treatment initiation (week 0);
- T3: assessment during the third week of radiotherapy (week 3);
- T6: assessment at the completion of radiotherapy (week 6);
- T9: post-treatment follow-up assessment performed three weeks after radiotherapy completion (week 9).

At each timepoint, oral mucositis severity was evaluated using CTCAE and OMAS, swallowing-related quality of life was assessed using the MDADI questionnaire, and body weight was recorded.

### 2.4. Statistical Analysis

Descriptive statistics were used to summarize patient demographics, treatment characteristics, and longitudinal clinical findings.

Given the retrospective exploratory design and the limited sample size, the analysis focused on the assessment of associations between oral mucositis severity and clinical outcome measures rather than hypothesis-driven efficacy testing.

Correlations between OMAS scores and clinician-reported toxicity (CTCAE), patient-reported swallowing-related quality of life (MDADI global and composite scores), and body weight were evaluated at each predefined assessment timepoint using Spearman’s rank correlation coefficient, given the non-parametric distribution of the variables.

A two-sided *p*-value < 0.05 was considered statistically significant. Because of the exploratory nature of the study, no adjustment for multiple comparisons was applied. Therefore, *p*-values should be interpreted cautiously and the results considered hypothesis-generating.

### 2.5. Ethical Considerations

Institutional Review Board Statement: Ethical approval was not required because this retrospective observational study was based exclusively on anonymized clinical data collected during routine clinical practice and did not involve any intervention beyond standard patient care. The study was conducted in accordance with local institutional policies and applicable regulations governing retrospective observational research.

Informed Consent Statement: Written informed consent was obtained from all patients for treatment and for the use of anonymized clinical data for research purposes. Additional written informed consent was obtained for the publication of anonymized clinical and endoscopic images included in this article.

## 3. Results

### 3.1. Patient Characteristics

Fifty-four consecutive patients were screened. Thirty-two fulfilled the eligibility criteria and had complete longitudinal assessments available for analysis. Baseline demographic, clinical, and treatment characteristics are summarized in Table 1. The median age was 66 years, and most patients were male (68.8%). The most frequent primary tumor sites were the oral cavity, larynx, and oropharynx. Most patients had squamous cell carcinoma and stage III–IV disease. Radiotherapy was delivered in both definitive and adjuvant settings, with concurrent chemotherapy administered in 62.5% of cases. At baseline (T0), oral mucositis was absent or minimal in nearly all patients, as expected before treatment initiation.

### 3.2. Longitudinal Evolution of Oral Mucositis

All patients developed treatment-related oral mucositis during radiotherapy. Mucositis severity progressively increased during treatment, reaching the highest OMAS values at T6, followed by partial improvement and subsequent resolution during follow-up.

**Figure 2:**
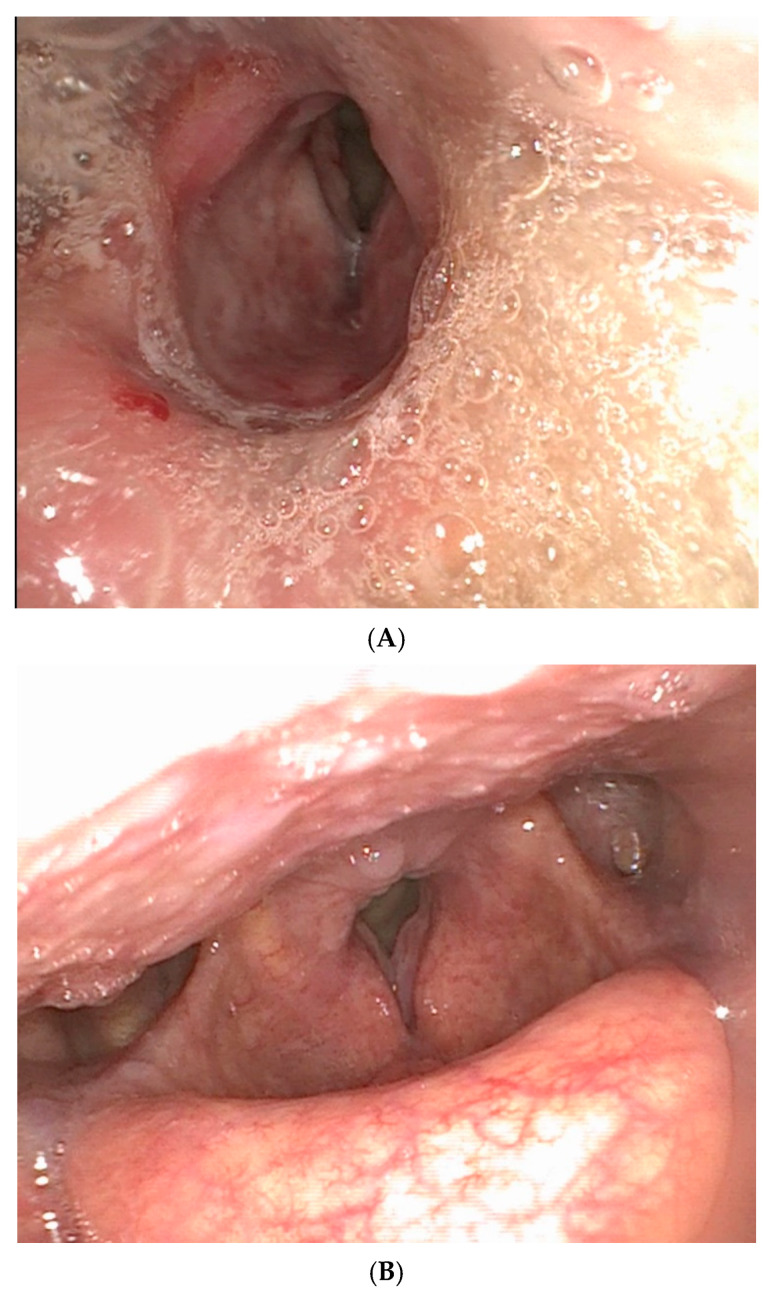
Representative endoscopic images obtained during routine otorhinolaryngological follow-up, showing treatment-related pharyngolaryngeal mucosal injury at T3 (**A**) and partial resolution at T9 (**B**). Written informed consent for publication of these anonymized images was obtained from the patient.

Representative clinical images illustrating peak mucosal toxicity and subsequent improvement are shown in Figure 2.

### 3.3. Correlation Between Oral Mucositis Severity and Clinical Outcomes

#### 3.3.1. Objective and Clinician-Reported Toxicity

At T3, oral mucositis severity assessed by OMAS demonstrated a moderate positive correlation with clinician-reported toxicity according to CTCAE (ρ = 0.52, *p* = 0.0028).

No statistically significant correlations between OMAS and CTCAE were observed at later assessment timepoints.

#### 3.3.2. Patient-Reported Functional Outcomes

Clinician-reported toxicity and objective mucosal assessment showed a similar temporal pattern, with severity progressively increasing from baseline (T0) to treatment completion (T6), followed by partial recovery at the post-treatment follow-up assessment (T9).

Higher OMAS scores were significantly associated with worse swallowing-related quality of life.

Mucosal severity progressively increased during treatment, with the highest median OMAS value observed at T6, corresponding to treatment completion. However, the strongest correlations between OMAS and CTCAE/MDADI were observed at T3, suggesting that clinician-reported toxicity and patient-reported swallowing impairment may become clinically evident before the maximum objective mucosal burden is reached.

At treatment completion, the inverse association remained statistically significant for the MDADI composite score (ρ = −0.41, *p* = 0.022), while no statistically significant correlation was observed for the global score.

During follow-up, no statistically significant associations were identified between residual mucosal findings and patient-reported swallowing outcomes.

#### 3.3.3. Nutritional Status

Information regarding enteral nutritional support was available for all patients. Fourteen patients (43.8%) required percutaneous endoscopic gastrostomy (PEG) placement during treatment, while eight patients (25.0%) required temporary nasogastric tube (NGT) support. The remaining ten patients (31.2%) maintained oral intake without the need for enteral nutritional support.

**Figure 3:**
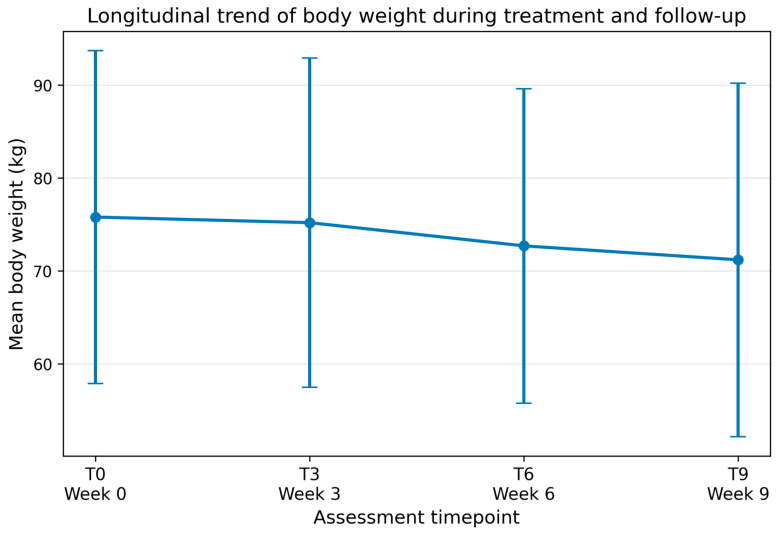
Longitudinal trend of body weight during treatment and follow-up. Mean body weight was recorded at predefined weekly assessment timepoints (T0, T3, T6, and T9), demonstrating a progressive decline during radiotherapy with partial stabilization during follow-up.

Treatment interruptions were uncommon. One patient undergoing concurrent chemoradiotherapy experienced febrile neutropenia during the second week of treatment, requiring hospitalization and a 7-day interruption of therapy. A second patient undergoing concurrent chemoradiotherapy required a 5-day treatment interruption during the third week because of severe oral mucositis. No permanent treatment discontinuations were observed. Mean body weight decreased from 75.8 ± 17.9 kg at baseline (T0) to 71.2 ± 19.0 kg at T9, corresponding to an absolute mean reduction of 4.6 kg and an approximate relative decrease of 6.1% from baseline. Weight loss was modest at T3 (−0.8%), became more pronounced at T6 (−4.1%), and reached −6.1% at T9. No statistically significant correlations were observed between OMAS scores and body weight at any predefined assessment timepoint. As illustrated in Figure 3, mean body weight progressively decreased throughout radiotherapy, with stabilization observed during the early post-treatment follow-up period.

### 3.4. Summary of Correlation Analyses

A summary of the correlation analyses between oral mucositis severity and clinical outcome measures is reported in Table 2.

Overall, the strongest associations were observed during the acute treatment phase, when objective mucosal injury was associated with both clinician-reported toxicity and patient-reported functional impairment.

The longitudinal evolution of oral mucositis severity, swallowing-related quality of life, and body weight is summarized in Table 3. OMAS scores progressively increased during treatment, reaching peak values at T6, followed by substantial improvement at T9. Similarly, MDADI scores worsened during treatment and partially recovered during follow-up, while body weight showed a progressive decline throughout the observation period.

## 4. Discussion

Oral mucositis remains one of the most frequent and clinically relevant acute toxicities in patients undergoing radiotherapy or chemoradiotherapy for head and neck cancer, with substantial implications for symptom burden, nutritional status, treatment adherence, and quality of life [5,8,9,10,11]. Although oral mucositis is routinely assessed in clinical practice, clinician-reported toxicity grading may not fully capture the multidimensional burden experienced by patients.

In the present retrospective observational study, we performed a longitudinal multidimensional assessment integrating clinician-reported toxicity (CTCAE), objective mucosal damage (OMAS), patient-reported swallowing-related quality of life (MDADI), and body weight changes in a real-world cohort of patients receiving radiotherapy-based treatment for head and neck cancer.

Kariosyte^®^ was included in the routine supportive-care protocol adopted at our institution because it contains ingredients commonly employed for oral mucosal protection and hydration during radiotherapy. Since all patients received the same supportive-care approach and no comparator group was available, the present study was not intended to assess the efficacy of this intervention [7,12,13,14,15,16,17,18,19,20].

Our findings confirm the dynamic temporal evolution of treatment-related oral mucositis, with progressive worsening during treatment, peak severity during active therapy, and subsequent improvement after treatment completion. This pattern is consistent with the established biological and clinical course of radiation-induced mucosal injury [5,6,7].

A key finding of the present study is the observation that objective mucosal injury assessed by OMAS showed a moderate correlation with clinician-reported toxicity at peak treatment burden, while also demonstrating a significant inverse relationship with patient-reported swallowing-related quality of life. These findings suggest that objective mucosal assessment may provide clinically relevant information that is not fully captured by clinician-based toxicity grading alone [14,15,16].

Patient-reported outcomes are increasingly recognized as essential complementary tools in oncology, particularly in head and neck cancer, where acute treatment-related symptoms directly affect swallowing, oral intake, communication, and social functioning [17,18]. In our cohort, worse objective mucosal injury was associated with poorer MDADI scores during the period of highest acute toxicity, reinforcing the relationship between visible mucosal damage and patient-perceived functional impairment.

The longitudinal body weight analysis further supports the broader functional relevance of oral mucositis [19,20,21,22]. Although no statistically significant direct correlation was observed between OMAS scores and body weight at individual timepoints, patients demonstrated progressive weight loss during treatment, likely reflecting the combined effects of oral pain, dysphagia, reduced oral intake, and cumulative treatment burden [23,24,25].

From a clinical perspective, these findings support the use of multidimensional toxicity assessment strategies integrating clinician-reported toxicity, objective mucosal evaluation, and patient-reported outcomes [26,27]. Such an approach may provide a broader characterization of treatment-related toxicity and patient experience [28,29].

All patients in this cohort received supportive care according to routine institutional clinical practice throughout treatment, including oral hygiene measures, symptomatic management, nutritional monitoring, and supportive interventions as clinically indicated. Because of the retrospective observational design and the absence of a comparator group, the present study was not intended to evaluate the efficacy of any specific supportive intervention, and no causal inferences regarding individual supportive care measures can be drawn.

The partial dissociation between mucosal injury and patient-reported swallowing outcomes may reflect the multifactorial nature of dysphagia, which is influenced not only by mucosal damage but also by pain perception, edema, xerostomia, muscular dysfunction, and adaptive coping mechanisms.

The lack of a significant correlation between OMAS and body weight should be interpreted cautiously. Absolute body weight is strongly influenced by baseline body size and may not fully reflect treatment-related nutritional deterioration. In the present cohort, mean body weight decreased by approximately 6.1% from baseline to T9. Furthermore, nutritional support interventions, including PEG placement in 14 patients and temporary nasogastric tube support in 8 patients, may have attenuated weight loss and confounded the relationship between mucositis severity and nutritional outcomes.

Several limitations should be acknowledged. First, the retrospective design introduces the possibility of selection bias and limits causal interpretation. Second, the relatively small sample size restricts statistical power and limits generalizability. Third, the clinical heterogeneity of the cohort, including differences in tumor subsite and treatment setting, may have influenced toxicity burden and functional outcomes. Patients with laryngeal cancer may have experienced a different oral mucosal dose distribution compared with patients with oral cavity or oropharyngeal tumors. Finally, the exploratory correlation-based statistical approach should be considered hypothesis-generating rather than confirmatory. Given the limited sample size, mixed-effects or multivariable longitudinal models were not considered sufficiently robust.

In addition, multiple correlation analyses were performed without adjustment for multiplicity. Consequently, the risk of type I error cannot be excluded, and the findings should be interpreted as exploratory.

A further limitation is the exclusion of patients with incomplete longitudinal assessments. Because serial otorhinolaryngological evaluations were required to obtain repeated OMAS and CTCAE measurements, patients who missed scheduled follow-up visits could not be included in the final analysis. This may have introduced selection bias and should be considered when interpreting the results.

The study follow-up was intentionally limited to T9 because the primary objective was to evaluate the onset, peak severity, and early resolution of acute radiation-induced oral mucositis. According to published evidence [30], oral mucositis generally resolves within the first month after radiotherapy completion. Although all patients continued their scheduled oncological follow-up thereafter, subsequent visits were not included in the analysis because acute mucosal toxicity was no longer the primary focus of assessment.

Despite these limitations, this study provides a clinically relevant real-world characterization of oral mucositis burden in patients undergoing radiotherapy-based treatment for head and neck cancer. Prospective studies with larger and more homogeneous cohorts are warranted to validate these observations and to further refine multidimensional supportive care monitoring strategies.

## 5. Conclusions

In conclusion, this study identified significant associations between clinician-reported toxicity (CTCAE), objective mucosal assessment (OMAS), and patient-reported swallowing outcomes (MDADI) in patients undergoing radiotherapy-based treatment for head and neck cancer. These findings suggest the potential usefulness of a multidimensional assessment approach for characterizing treatment-related oral mucositis burden. Given the exploratory nature of this study and the limited sample size, these observations should be considered hypothesis-generating and require confirmation in larger prospective studies.

## Figures and Tables

**Table 1:** Clinical and Treatment Characteristics. Study population (*n* = 32) [4].

Variable	Category	*n* (%)
Tumor subsite		
	Oral cavity	10 (31.2)
	Larynx	9 (28.1)
	Oropharynx	6 (18.7)
	Parotid gland	2 (6.3)
	Submandibular gland	2 (6.3)
	Hypopharynx	2 (6.3)
	Maxillary sinus	1 (3.1)
Stage		
	III	20 (62.5)
	IVA	9 (28.1)
	IVB	3 (9.4)
Histology		
	Squamous cell carcinoma	25 (78.1)
	Adenocarcinoma	5 (15.6)
	Other	2 (6.3)
Grading		
	G3	20 (62.5)
	G2	11 (34.4)
	G1	1 (3.1)
Treatment setting		
	Adjuvant CRT	10 (31.2)
	Definitive CRT	10 (31.2)
	Adjuvant RT	8 (25.0)
	Definitive RT	4 (12.5)
Concomitant chemotherapy		
	Yes	20 (62.5)
Weekly cisplatin		19
Weekly cetuximab		1
	No	12 (37.5)
Metastatic disease at baseline		
	No	32 (100.0)
Age		
	Median	66 (34–83 years)
Sex		
	Male	22 (68.8)
	Female	10 (31.2)

**Table 2:** Correlation between oral mucositis severity (OMAS) and clinical variables.

Timepoint	Variable	Spearman ρ	*p*-Value
T3	CTCAE mucositis	0.52	0.0028
	MDADI Global score	−0.37	0.038
	MDADI Composite score	−0.50	0.0045
	Body weight	−0.23	0.22
T6	CTCAE mucositis	0.28	0.12
	MDADI Global score	−0.29	0.12
	MDADI Composite score	−0.41	0.022
	Body weight	−0.03	0.86
T9	MDADI Global score	−0.22	0.22
	MDADI Composite score	−0.29	0.11

Correlation between oral mucositis severity (OMAS) and clinical variables. Spearman’s rank correlation coefficients (ρ) were calculated to evaluate associations between OMAS scores and CTCAE grade, body weight, and MDADI scores at each assessment timepoint. Positive coefficients indicate a direct association with increasing OMAS scores, whereas negative coefficients indicate an inverse association.

**Table 3:** Longitudinal evolution of oral mucositis severity, swallowing-related quality of life, and body weight across predefined assessment timepoints.

Variable	T0	T3	T6	T9
OMAS score, median (IQR)	0 (0–0)	2 (0–4)	3 (0–4.5)	0 (0–2)
MDADI Global score, mean ± SD	3.7 ± 1.3	3.5 ± 1.2	3.5 ± 1.2	3.6 ± 1.4
MDADI Composite score, mean ± SD	77.8 ± 18.5	73.6 ± 19.0	69.5 ± 20.0	73.1 ± 19.7
Body weight (kg), mean ± SD	75.8 ± 17.9	75.2 ± 17.7	72.7 ± 16.9	71.2 ± 19.0
Weight change from baseline (%)		−0.8	−4.1	−6.1

Abbreviations: OMAS, Oral Mucositis Assessment Scale; MDADI, MD Anderson Dysphagia Inventory; SD, standard deviation; IQR, interquartile range.

## Data Availability

The original contributions presented in this study are included in the article. Further inquiries can be directed to the corresponding author.
